# OvaRePred (HerTempo): an enhanced ovarian aging clock for personalized reserve assessment, endocrine age modeling, and predicting reproductive milestones across the female lifecycle

**DOI:** 10.3389/fendo.2025.1658068

**Published:** 2025-09-12

**Authors:** Huiyu Xu, Guoshuang Feng, Rui Yang, Yong Han, Hongbin Chi, Rong Li

**Affiliations:** ^1^ State Key Laboratory of Female Fertility Promotion, Center for Reproductive Medicine, Department of Obstetrics and Gynecology, Peking University Third Hospital, Beijing, China; ^2^ National Clinical Research Center for Obstetrics and Gynecology (Peking University Third Hospital), Beijing, China; ^3^ Key Laboratory of Assisted Reproduction (Peking University), Ministry of Education, Beijing, China; ^4^ Beijing Key Laboratory of Reproductive Endocrinology and Assisted Reproductive Technology, Beijing, China; ^5^ National Clinical Key Specialty Construction Program, China (2023), Beijing, China; ^6^ Big Data Center, Beijing Children’s Hospital, Capital Medical University, National Center for Children’s Health, Beijing, China; ^7^ Department of Thoracic Surgery, Zhejiang Provincial People’s Hospital, Affiliated People’s Hospital, Hangzhou Medical College, Hangzhou, Zhejiang, China

**Keywords:** ovarian reserve, anti-müllerian hormone (AMH), ovarian aging curve, endocrine age, reproductive milestone prediction, personalized fertility planning, health management

## Abstract

**Background:**

Women display marked variability in ovarian reserve, which is pivotal for fertility and menopausal timing. Traditional criteria, such as Bologna and Poseidon, classify women into broad groups but do not provide individualized predictions for ovarian aging or reproductive milestones. This study aims to refine the AA model (AMH + age) to enhance clinical usability, robustness, and interpretability.

**Materials and Methods:**

Single-center retrospective ART cohort (GnRH-antagonist cycles, 2017–2021). Training: 15,241 cycles (2017–2019); Testing: 14,498 cycles (2020–2021). Poor ovarian response (POR) was defined as <5 oocytes. Three logistic-regression specifications were compared: categorical (Model-0), continuous (Model-1), and polynomial (age quadratic, AMH cubic; Model-2). Discrimination (AUC), calibration, and net reclassification improvement (NRI) were evaluated. A two-parameter logistic curve was fitted to age versus predicted POR (used population-level as “predicted DOR”) to construct an ovarian-aging trajectory and derive an interpretable “endocrine-age” index. Sensitivity analyses assessed cycle-day AMH variation; a community dataset was used to compare age-stratified AMH distributions.

**Results:**

While all models achieved comparable discrimination (AUC ≈ 0.85), a cubic transformation model (Model-2) demonstrated superior calibration and was selected as the final algorithm. A two-parameter logistic curve allowed translation of ovarian reserve scores into an “endocrine age” and enabled individualized prediction of future milestones, such as diminished reserve with ovarian score of 50 and perimenopause, the lowest ovarian reserve score in our ART population. AMH sampling on different cycle days showed only modest effects from minor fluctuations; only substantial AMH decreases significantly affected prediction accuracy. Age-stratified AMH distributions were similar between ART and community cohorts in women <40, supporting external relevance. The updated OvaRePred (HerTempo) model is cost-effective, scalable, and operationally simple.

**Conclusion:**

OvaRePred (HerTempo) delivers individualized, well-calibrated estimates of ovarian reserve and an interpretable endocrine-age index and future fertility milestone onset. While the tool can inform personalized fertility planning and may have broader public-health utility, the algorithm is trained on ART endpoints. Any projections of future reproductive milestones derived from the population ovarian-aging curve—and the fixed-interval hypothesis that underpins that curve—are hypothesis-generating and require prospective validation, particularly in non-ART cohorts with longitudinal follow-up.

## Introduction

As is well known, women lose their fertility at menopause due to the depletion of ovarian reserve, which is widely considered the most critical factor affecting fertility (1, 2). However, what is less appreciated is the significant inter-individual variability in ovarian reserve (3, 4). Ovarian reserve, defined by the number of primordial follicles, exhibits marked differences even at birth, from thousands to millions (1). Although the rate of ovarian aging is relatively consistent among individuals, these inherent variations in ovarian reserve lead to a wide disparity in the age at menopause (1, 5). The Bologna (6) and Poseidon (7) criterias for poor ovarian response, although commonly used for assessing ovarian reserve, have notable limitations. Specifically, the Bologna standard categorizes patients into only two groups, while the Poseidon criteria, despite subdividing into four categories, still do not fully capture individual differences. Additionally, both standards heavily rely on ultrasound-based antral follicle count (AFC) assessments, which typically require transvaginal ultrasound for greater accuracy. This method, however, can cause discomfort for some women and is subject to variability due to differences in equipment and operator proficiency. Moreover, they assess ovarian reserve only at a single point in time without providing dynamic predictions of future changes. In light of these deficiencies, and given the vast individual differences in ovarian reserve, the ability to perform individualized ovarian reserve assessments and to predict reproductive milestones is of paramount importance.

In 2023, the reproductive center team at Peking University Third Hospital leveraged their extensive big data resources to introduce the OvaRePred (HerTempo) tool for ovarian reserve evaluation and future milestone prediction (8, 9). This tool has since been adopted in multiple hospitals across China, including Peking University Third Hospital, as well as in health examination centers and online platforms, with favorable patient feedback. OvaRePred (HerTempo) provides a ranking of ovarian reserve from optimal to poor based on the probability of a poor ovarian response (POR), generating a score for current ovarian reserve. Furthermore, by integrating the Fixed Interval theory—which posits that the ovarian aging curves of populations are similar and follow an S-shaped curve—our team has constructed a population-level ovarian aging curve. This enables the tool not only to assess current ovarian reserve but also to predict the age at which critical reproductive milestones occur, such as the onset of diminished ovarian reserve (DOR, corresponding to an ovarian reserve score of 50) and the beginning of perimenopause (marked by the lowest ovarian reserve score in our ART populations).

OvaRePred (HerTempo) proposes three prediction models tailored to different clinical scenarios—AAFA (AMH-AFC-FSH-Age), AFA (AMH-FSH-Age), and AA (AMH-Age)—for evaluating ovarian reserve and forecasting subsequent reproductive milestones. Although these models incorporate different combinations of variables, their predictive performance is statistically similar, suggesting that a simpler model may suffice in clinical practice. In particular, the AA model, which relies solely on anti-Müllerian hormone (AMH) and age, offers the advantages of simplicity and cost-effectiveness while maintaining high predictive accuracy (10). Moreover, AMH measurement is not time-restricted and blood can be drawn on any day of the menstrual cycle, the AA model offers greater flexibility and convenience. However, both the AFA and AAFA models depend on FSH measurements obtained via precisely timed blood draws during the menstrual cycle, and the AAFA model further requires antral follicle counts by transvaginal ultrasound—adding procedural complexity, higher costs, and greater patient discomfort. Thus, our current study focuses on updating and refining the AA model using larger datasets and advanced statistical techniques, with the ultimate goal of establishing a robust, high-performing ovarian reserve assessment and reproductive milestone prediction tool that is both user-friendly and operationally efficient.

Our original AA model (10) employed categorical transformations for its independent variables. Categorical variable models offer simplicity and ease of interpretation by grouping data to capture overall trends, maintain stability, and resist the influence of outliers (11, 12). While this method effectively captures general trends and ensures stability, it falls short in reflecting finer changes. In contrast, continuous variable models retain the complete spectrum of the original data and, through appropriate transformations (such as a cubic transformation), can more accurately capture the nonlinear relationships between variables. This leads to enhanced discriminative power and improved predictive accuracy. However, continuous models are more complex to construct and interpret, are more sensitive to outliers, and may risk overfitting, particularly with smaller sample sizes (12). For these reasons, we aim to update our AA model by exploring various transformations of the independent variables. Our goal was to achieve better discrimination and calibration compared to the original model.

Despite its clinical promise, the original AA model—which categorizes AMH and age—may be too coarse to capture subtle nonlinear relationships, and its calibration can be further improved. Meanwhile, although the AFA and AAFA models achieve similar discrimination, their reliance on cycle−timed FSH measurements and transvaginal AFC assessment increases complexity, cost, and patient burden. To address these gaps, the present study leverages a substantially expanded single−center ART dataset to explore different transformations of AMH and age, evaluate the variations of AMH on model performance, and rigorously compare model discrimination, calibration, and reclassification performance. Our aim is to refine the AA model into a streamlined, high−precision tool that maintains interpretability and operational efficiency, thereby enhancing personalized ovarian reserve assessment, endocrine age evaluation and the prediction of key reproductive milestones.

## Materials and methods

### Study population and data sources

This retrospective, single-center analysis enrolled women undergoing controlled ovarian stimulation with gonadotropin-releasing hormone (GnRH) antagonist protocols from 2017 to 2021. All participants received stimulation at our center, with serum AMH levels, chronological age, and pertinent clinical variables recorded at baseline. No apparent outliers in AMH or age were identified in the exported database; thus, no data exclusion for outliers was performed. Retaining the full spectrum of observed AMH and age values enhances the model’s generalizability, particularly for individuals at the extremes of ovarian reserve. Initially, 16,327 antagonist cycles from January 2017 to December 2019 constituted the training cohort, and 15,596 cycles from January 2020 to December 2021 comprised the independent validation cohort. After removing multiple cycles contributed by the same women to address the clustering issue, the final numbers of cycles included in the analysis were 15,241 for 2017–2019 and 14,498 for 2020–2021. Poor ovarian response (POR) was defined as retrieval of fewer than five oocytes. As this study aims to update our previous AA model, **Table 1** reports only the distributions of AMH and age according to POR status. This study was approved by the Institutional Review Board of Peking University Third Hospital (approval number: 2015sz-017).

**Table 1:** Characteristics of the modeling dataset and the external validation dataset.

Variable	Statistic	Training set (2017-2019)		Test set (2020-2021)	
		POR=No	POR=Yes	POR=No	POR=Yes
N		13017	2224	12468	2030
AMH (ng/ml)	Median	3.14	0.67	2.79	0.75
	25^th^ percentile	1.7	0.34	1.58	0.4
	75^th^ percentile	5.4	1.31	4.69	1.37
Age (years)	Median	32	36	32	36
	25^th^ percentile	29	32	30	32
	75^th^ percentile	35	40	35	39

POR, poor ovarian response, with less than 5 oocytes retrived.

### Clarification of POR and DOR definitions

To avoid ambiguity, we clarify the distinct definitions of poor ovarian response (POR) used in this study. Clinically, POR was defined as retrieval of fewer than five oocytes, and this criterion was applied consistently for all modeling and validation analyses. For constructing the ovarian aging curve, we defined predicted POR (or diminished ovarian reserve, DOR) using a probability threshold of 0.15, reflecting the actual incidence of POR in our cohort, rather than the standard 0.5 binary classification threshold used in the software. We recognize that these differing definitions may cause confusion; therefore, we emphasize that the clinical definition (fewer than five oocytes) is used for model evaluation, while the 0.15 threshold is employed specifically for population-level aging curve construction.

### Statistical analysis

A logistic regression framework was used to model the risk of POR (< 5 oocytes retrieved), facilitating direct comparison with our previously published model (Model-0). Three candidate specifications for age and AMH were evaluated:

1. Model-1 (continuous): age and AMH were included as untransformed continuous variables.
2. Model-2 (polynomial): age was represented by a quadratic term and AMH by a cubic term, based on exploratory generalized additive modeling.

Model discrimination was quantified by the area under the receiver operating characteristic curve (AUC). Calibration was assessed via calibration plots comparing predicted probabilities with observed POR rates. Incremental improvement over Model-0 was measured using the net reclassification improvement (NRI) index.

Subsequently, the relationship between the proportion of predicted POR (also defined as predicted DOR in our study) and age was characterized. Given the anticipated sigmoidal pattern, a logistic growth curve was fitted to the age-DOR data.

To evaluate whether the AMH–age model derived from assisted reproductive technology (ART) data is generalizable to a broader population, we conducted an exploratory comparison of AMH distributions between our single-center ART cohort and a community-based epidemiologic survey^13^. Participants in both datasets were stratified into three age groups: ≤30 years, >30–≤40 years, and >40 years. For each age group, we plotted the empirical distribution of serum AMH concentrations and compared their medians and interquartile ranges.

All analyses were conducted in JMP Pro 17.0 (SAS Institute) and R 4.4.1, with statistical significance defined as two-sided p < 0.05.

## Results

### Ovarian reserve scoring construction

#### Prediction of poor ovarian response

This study aims to optimize the original AA model (10)—which utilizes AMH and age to predict the probability of POR—by expanding the sample size and performing a comprehensive analysis and appropriate transformation of the independent variables (AMH and age). Three distinct models were developed: the original model with categorical transformation of independent variables (Model-0), a new model without any transformation (Model-1), and a new model employing a cubic transformation of AMH and a quadratic term of age, based on exploratory generalized additive modeling. (Model-2).

**Figure 1:**
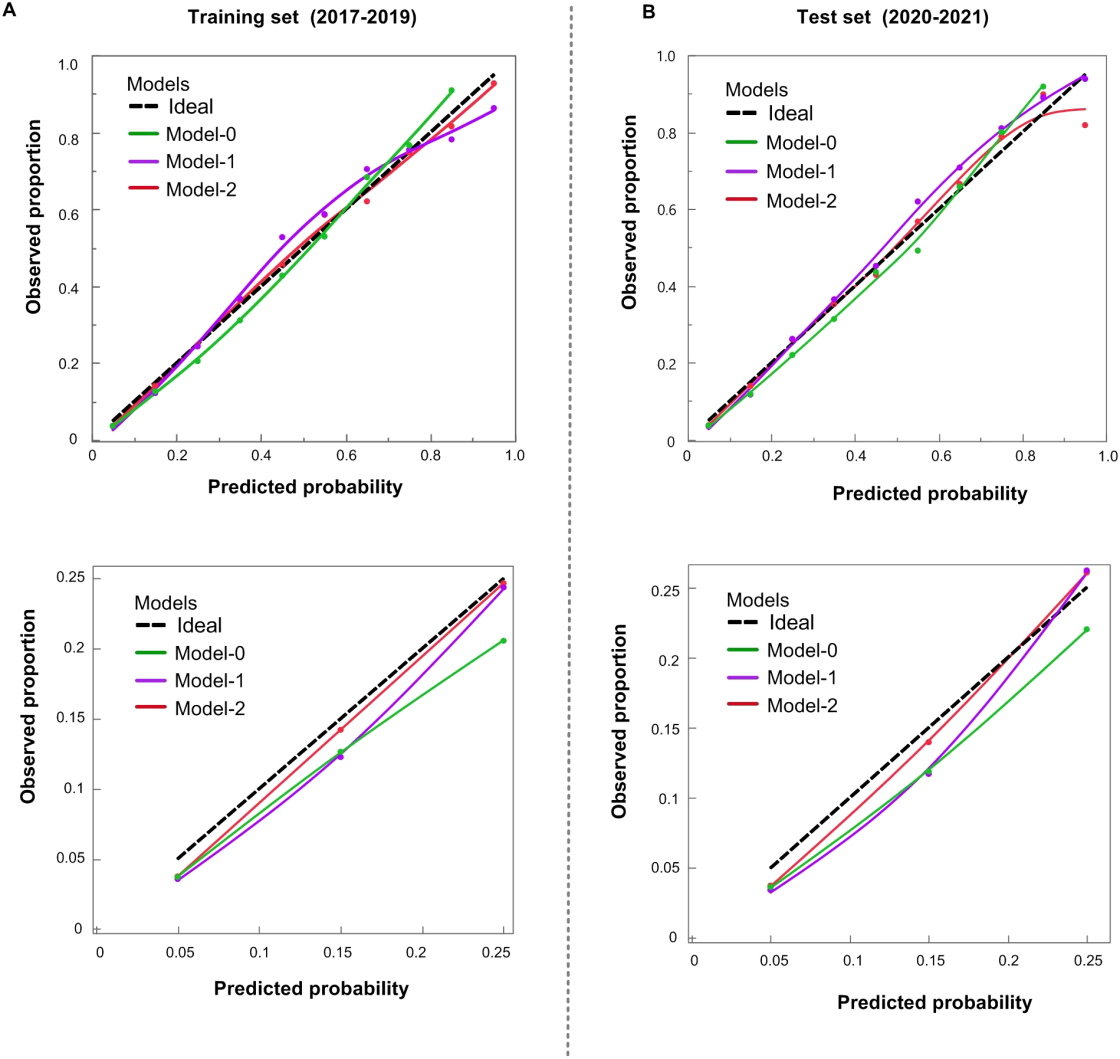
Calibration of POR prediction models. **(A)** Training cohort (2017–2019); **(B)** Test cohort (2020–2021). Upper panels cover the full 0–1.0 range; lower panels zoom to 0–0.25. Dashed black = ideal. Model−0 (green) overestimates risk < 0.60; Model−1 (purple) improves fit; Model−2 (red) tracks the ideal line most closely.

Comparisons using NRI, ROC curves (**Table 2**), and calibration curves (**Figure 1**) led to the following key conclusions. While differences in ROC and NRI metrics were not markedly significant, the calibration curve of Model-2 was superior to the other models. **Figure 1** compares the calibration of Model-0, Model-1, Model-2, and the Ideal model across both the training (2017–2019) and test (2020–2021) datasets. Model-2 consistently performs the best, with its curve closely aligning with the Ideal model, especially in the mid and low probability ranges. Although Model-1 also performs well, Model-0 shows significant deviations compared to the other models. The lower panel zooms in on the 0-0.25 probability range, which is particularly important as it represents 82.3% of the female population in our dataset. In this zoomed-in view, Model-2 again outperforms the other models, demonstrating superior calibration and making it the most reliable for predicting outcomes in this most common probability range.

**Table 2:** Discrimination performance for the four models predicting poor ovarian response (POR).

Names of models	AUC (95% CI) in training set	AUC (95% CI) in test set	NRI in training set	NRI in test set
Model-0	0.858 (0.849,0.867)	0.845 (0.834,0.855)	—	—
Model-1	0.860 (0.851,0.869)	0.847 (0.837,0.857)	0.0075 (-0.009,0.0239)	-0.0015 (-0.0179,0.015)
Model-2	0.861 (0.851,0.870)	0.847 (0.837,0.857)	0.004 (-0.0153,0.0233)	0.0128 (-0.0055,0.0312)

NRI, Net reclassification improvement.

Model−0: The original model, in which AMH and age were converted into categorical variables for modeling.

Model-1: Age and AMH were included as untransformed continuous variables.

Model-2: Age was represented by a quadratic term and AMH by a cubic term, based on exploratory generalized additive modeling.

Based on these findings, we have chosen the Model-2 as the final version of the updated model. In clinical practice, model calibration is of paramount importance. Good calibration ensures that the predicted incidence of POR accurately reflects the true incidence within the population, thereby guaranteeing that each predicted value closely approximates the actual occurrence rate. This accurate incidence estimation serves as a crucial foundation for subsequent predictions, such as the age at the onset of perimenopause.

### Ovarian reserve score conversion

In the OvaRePred (HerTempo) model, we convert the predicted probability of POR into an ovarian reserve score using the formula: (1 – predicted probability of POR) × 100. This conversion inversely maps the risk of POR to the quanlity of ovarian reserve: a higher score indicates a lower risk of POR and a better ovarian reserve, whereas a lower score suggests a higher risk of POR and a poorer ovarian reserve. This transformation not only standardizes the expression of the predicted probabilities but also makes the evaluation results more intuitive, thereby aiding clinicians in risk communication and decision-making.

### Ovarian aging curve and determination of endocrine age

**Figure 2:**
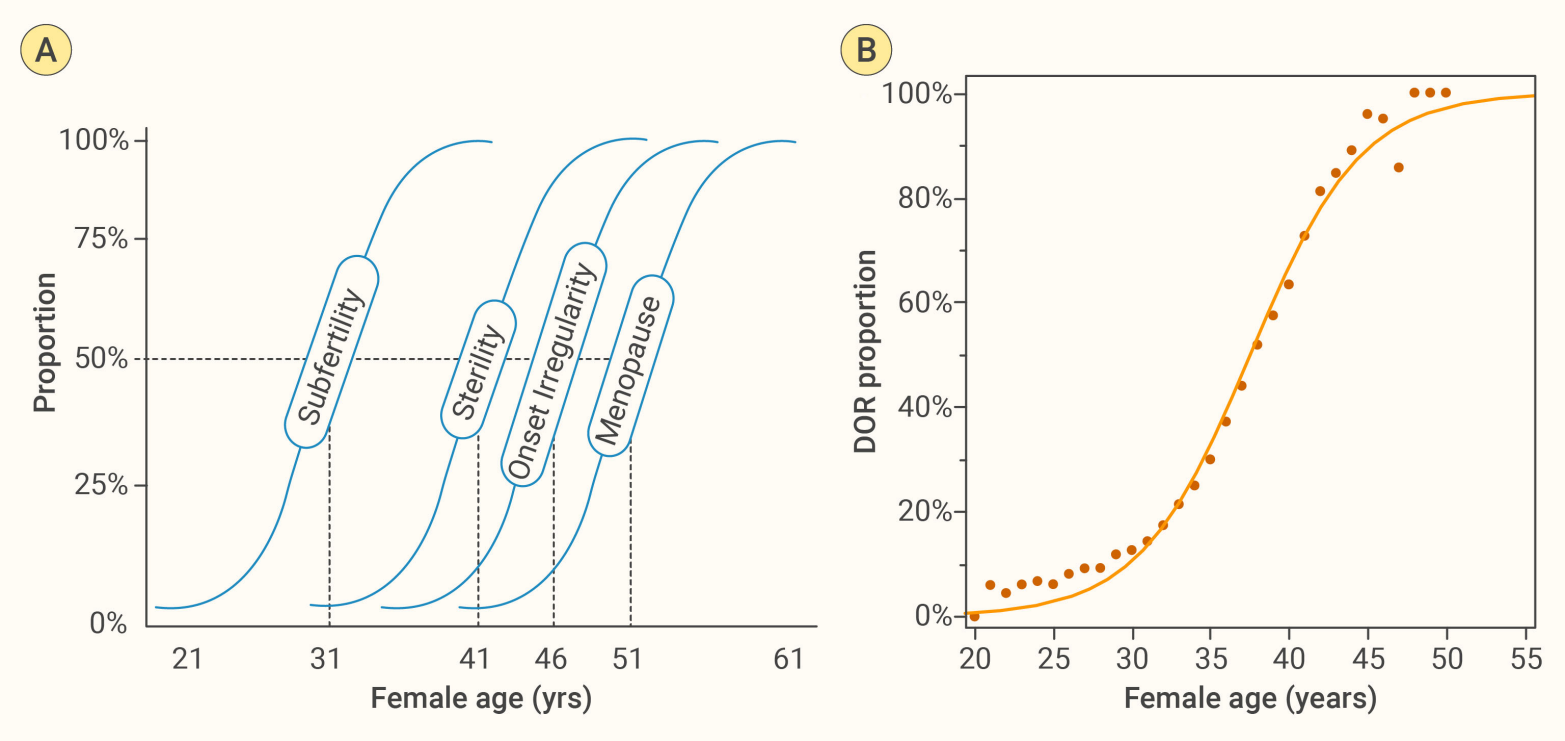
Age−related trajectory of female reproductive decline. **(A)** Fixed Interval Theory, from an early 20th−century cross−sectional study without birth control, proposes that age intervals between fertility states are relatively fixed (2, 5). We reconstructed its conceptual schematic by redrawing from the original reference, mapping four key milestones—subfertility, sterility, menstrual irregularity onset, and menopause—as sigmoidal functions of age. The 50% probability thresholds lie at approximately 31, 41, 46, and 51 years, respectively, illustrating a predictable, age−related progression of reproductive decline (5). **(B)** Empirical ovarian−aging curve derived from 31,923 first−cycle ART patients: observed proportions of diminished ovarian reserve (DOR, black dots) plotted against age with a two−parameter logistic fit. The close agreement confirms an S−shaped rise in DOR risk that underpins panel **(A)** schematic.

In our previous work, we constructed an ovarian aging curve using cross−sectional data based on the fixed−interval theory (2, 5), achieving an *r*² of 0.978 (8). In this study, we aim to further optimize that ovarian aging curve by incorporating more data and exploring additional methods. First, we recognize that ovarian aging follows a sigmoidal (S−shaped) trajectory. We therefore applied a two−parameter logistic curve to depict the trend in the predicted prevalence of POR across ages. DOR, predicted POR, was defined based on the newly developed Model-2, which applies a cubic transformation to AMH. Using an actual POR incidence rate of 0.15 as the cut-off to define POR, we plotted the proportion of DOR against age, as shown in **Figure 2**. The logistic curve model achieved an *r*² of 0.989—an improvement over the original 0.978—demonstrating superior overall fit. However, both the youngest and oldest age ranges show poorer alignment with the fitted curve, likely because fewer participants occupy these extremes, reducing the sample’s representativeness and statistical power there. In addition, increased biological variability and potential outliers at the tails can further weaken the model’s ability to capture true trends in those regions.

Using the fitted S−shaped curve, we can map any individual’s predicted probability of POR, also called predicted DOR in our study, onto the ovarian aging curve to derive their “endocrine age.” Here, ovarian endocrine age is an index that projects an individual’s ovarian reserve score onto the population ovarian aging trajectory, with the goal of reflecting the relative state of their ovarian function rather than their chronological age.

Specifically, by measuring AMH levels and noting the subject’s chronological age, we calculate a predicted probability of POR. We then locate that probability on the S−shaped ovarian aging curve constructed from large−scale population data; the age at which the curve reaches that probability is defined as the ovarian endocrine age. If a young woman’s ovarian reserve score corresponds to an endocrine age substantially higher than her chronological age, this may indicate accelerated ovarian decline and a potentially earlier drop in fertility. Conversely, if her endocrine age is close to or below her chronological age, it suggests her ovarian function is relatively well preserved. In this way, ovarian endocrine age reflects the true status of ovarian reserve and, compared with chronological age, more accurately reveals the degree and rate of ovarian aging.

### Predicting the age of onset for future fertility milestones

The “fixed-interval hypothesis” assumes that an individual’s ovarian aging trajectory shares the same shape as the population’s average curve—meaning the time intervals between successive stages of ovarian decline are constant, differing only in their starting points. In other words, although individuals may begin with different ovarian reserve levels, the overall pattern of functional decline over time can be described by a single sigmoid (S-shaped) curve (5). Evidence supporting this hypothesis primarily comes from cross-sectional observations (2). This hypothesis provides the theoretical basis and methodological support for predicting future ovarian reserve changes from a cross-sectional data.

**Figure 3:**
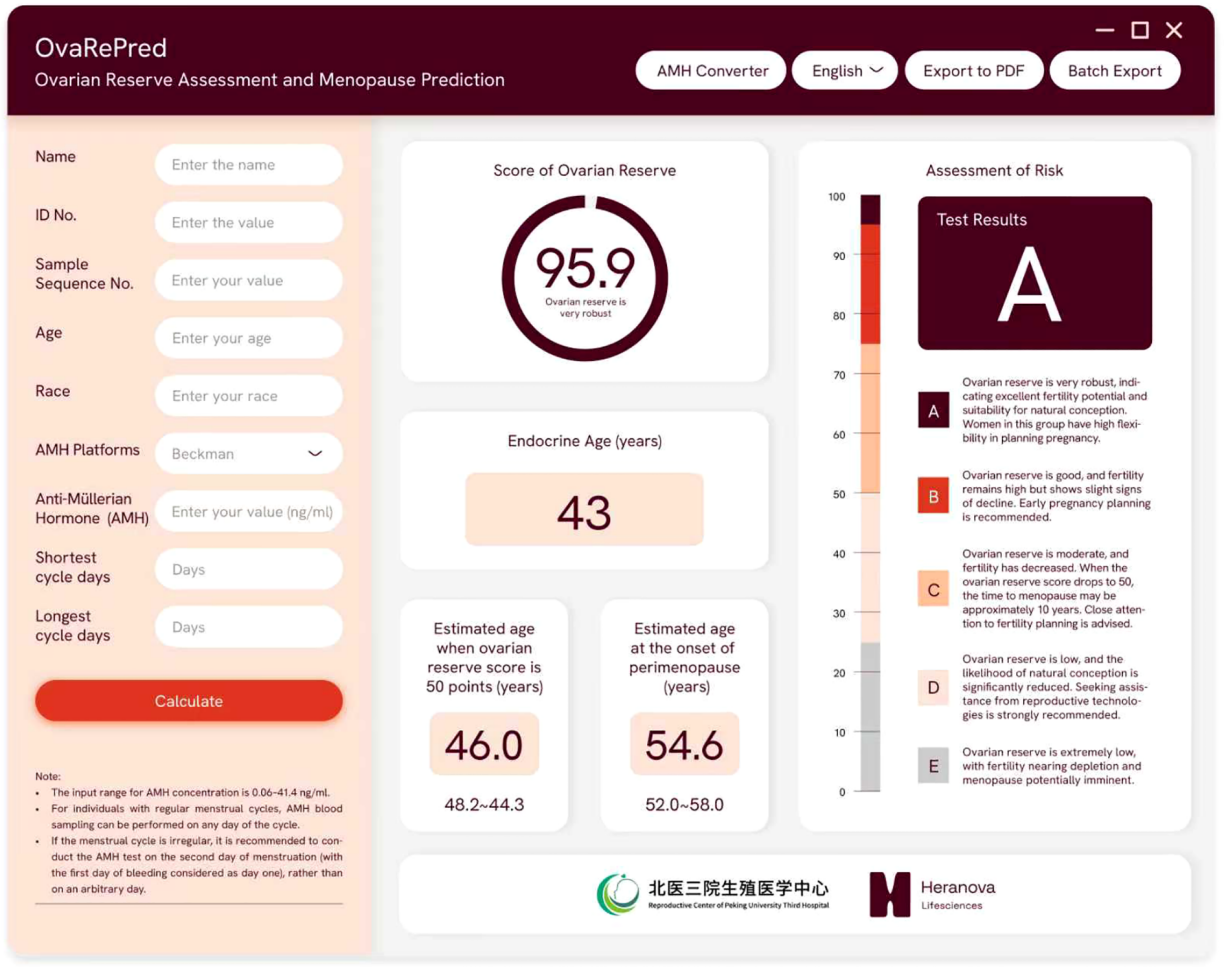
The OvaRePred software interface for ovarian reserve assessment and menopause prediction. The left panel captures patient demographics and AMH data, while the right panel presents outputs: ovarian reserve score with grade, endocrine ovarian age, estimated ages at 50−point reserve and perimenopause onset, and risk assessment.

On this basis, we derived the time intervals required for an individual to reach specific ovarian reserve states (predicted probability of POR). First, we map the subject’s current ovarian reserve score onto the S-curve to determine her position within the overall aging process. Next, using the fitted curve, we calculate the interval needed to drop from the current score to a predefined POR probability (e.g., a specific POR probability, which correspond to a specific ovarian reserve score), then converted these intervals into ages at which she would reach each milestone. We implemented this functionality in a software tool (see **Figure 3**). The program uses AMH and chronological age to calculate the user’s current reserve score and “endocrine age,” then predicts the ages at which she will reach a score of 50 and enter perimenopause (the lowest reserve score observed in an ART population). We have also updated these algorithms into an online tool (http://121.43.113.123:8005/).

### AMH distributions across age groups in ART vs. community populations

**Figure 4:**
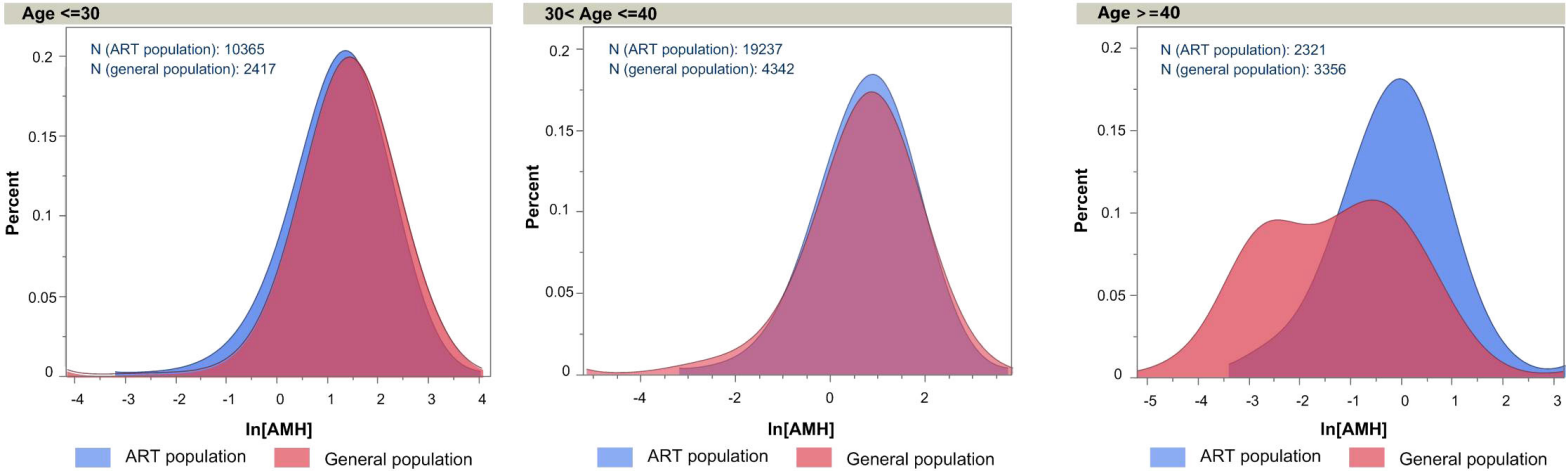
Age−specific log−transformed AMH distributions in ART versus general populations. Log−transformed AMH distributions for assisted−reproduction (ART, blue) and community−based general (red) populations stratified by age: ≤30 years (ART = 10 365, general = 2 417), 30–≤40 years (ART = 19 237, general = 4 342) and ≥40 years (ART = 2 321, general = 3 356). Distributions are nearly identical in younger groups; a divergence appears in women ≥40, reflecting differences in sample composition.

In our study, serum AMH concentrations from the ART cohort—comprising women undergoing assisted reproductive technology treatments—served as the primary data source. The median AMH values for women aged ≤ 30 years, > 30–≤ 40 years, and > 40 years were 3.65 ng/mL, 2.26 ng/mL, and 0.98 ng/mL, respectively. These were compared with median AMH levels reported in previously published population-based epidemiological studies representing the general female population, which showed values of 3.89 ng/mL, 2.28 ng/mL, and 0.34 ng/mL for the corresponding age groups (13). For women under 40, the empirical AMH distributions in the ART and general population cohorts were nearly identical, whereas in women over 40, the community data demonstrated a lower, left-shifted distribution (**Figure 4**), distributions are nearly identical in younger groups; a divergence appears in women ≥40, reflecting differences in sample composition. Likely reflecting the inclusion of a more representative cross-section of women in the general population survey. Since women under 40 made up the majority of both cohorts and exhibited highly similar AMH profiles, these findings indicate that the AMH–age relationship derived from our ART cohort is largely generalizable to the wider population. Detailed percentiles of AMH concentrations by age group for both cohorts are provided in **Supplementary Table 1**.

## Discussion

### Advancing ovarian reserve assessment: from categorical to continuous modeling in OvaRePred(HerTempo) optimization

Ovarian reserve is the primary determinant of female fertility (14). With advancing age or under disease impact, ovarian reserve gradually becomes depleted and fertility correspondingly declines until exhaustion. Assessing ovarian reserve enables early detection of insufficiency and supports personalized fertility planning and health management for women. This study aims to update and optimize the algorithms underlying the previous OvaRePred (HerTempo) tool using AA model (AMH+Age) (8, 10). The original AA model employed categorical transformations of its predictors. Categorical‐variable models are simple and interpretable: by grouping data they capture overall trends, maintain stability, and exhibit strong robustness to outliers. However, grouping can be crude, failing to reflect subtle variations, and the choice of cutpoints is often subjective.

In contrast, continuous‐variable models retain all original information and—through appropriate transformations (e.g., cubic terms)—more accurately characterize nonlinear relationships, enhancing discrimination and predictive accuracy. However, they are more complex to build and interpret, sensitive to outliers, and prone to overfitting with limited sample sizes. For these reasons, and given our earlier sample size, we converted continuous variables into categorical ones. In this update, however, with a larger dataset, we experimented with multiple predictor transformations, and the final Model-2 achieved superior calibration compared to the original Model-0.

### Comparison of parametric and non−parametric modeling approaches

During the modeling process, we explored various non-parametric methods (models without explicit functional forms), such as random forest, neural networks, and generalized additive models (GAMs). Although these models performed slightly better on the training set, their performance on the test set was similar to that of our traditional parametric models, with AUCs of 0.861, 0.863, and 0.863 for random forest, neural networks, and GAMs, respectively. This indicates that the non-parametric models achieved comparable results to the parametric approaches discussed in this manuscript. In comparison, we preferred parametric models with clear predictive formulas and high interpretability. These models have well-defined algorithmic principles that enable clinicians to intuitively understand the decision-making mechanisms, thereby improving trust and acceptance. Additionally, they are less demanding in terms of computational resources, making them easier to implement across different levels of healthcare institutions. Therefore, we ultimately selected a model that combines strong interpretability with practical clinical applicability.

### Impact of estradiol-driven AMH variation on model performance

During controlled ovarian stimulation, estradiol (E2) levels rise steadily, peaking around the hCG trigger day. Our supplementary data show that AMH concentrations decline significantly during this period—about 17.4% by day 6 and nearly 49.7% by the hCG day compared to day 2 levels (As shown in supplementary result). This sharp AMH decrease at hCG day leads to a notable drop in model performance, with the predictive accuracy (AUC) falling from 0.868 to 0.652. However, when AMH variation is minimal or moderate earlier in ovarian stimulation (e.g., cycle day 6 VS cycle day 2), model discrimination remains largely stable. These results highlight the importance of measuring AMH at the early follicular phase, when levels are more stable and E2 is low, to ensure optimal accuracy of ovarian reserve assessments and reproductive milestone predictions with tools like OvaRePred (HerTempo).

### Sources of AMH variability

When using the OvaRePred (HerTempo) tool, users should note that AMH is the primary and most heavily weighted predictive marker (8, 15), any element that causes AMH fluctuations may impact its prediction performance. Although AMH is generally considered stable during the menstrual cycle (16, 17), fluctuations in follicle status can lead to significant intra-cycle changes. AMH naturally declines with age (18), emphasizing the need for regular ovarian reserve assessments. Estrogenic drugs—such as those used in hormone replacement therapy or oral contraceptives—can temporarily lower AMH levels (19). Similarly, ovarian stimulation with FSH during assisted reproductive treatment (20), certain chemotherapy agents like cyclophosphamide (21), and acute ovarian conditions (e.g., cysts or inflammation) can affect AMH levels (22). Additional factors such as stress, systemic inflammation (23, 24), lifestyle changes like intense exercise or extreme diets (25), rapid weight fluctuations (26), testing time, blood collection techniques, sample storage conditions (27, 28), and variations between testing platforms or reagent batches (29) may all contribute to technical variability. Early pregnancy may cause a temporary decline in AMH (30), and short-term exposure to environmental endocrine disruptors (e.g., bisphenol A) can also affect AMH levels, either temporarily or permanently (31, 32).

### Standardization and best practices for AMH measurement

We recognize that the reliability of AMH-based predictive models depends critically on standardized measurement protocols and meticulous clinical documentation. Although AMH is generally considered relatively stable, both biological and technical factors—including cycle variability, medications, ovarian pathology, environmental exposures, and assay differences—can influence its levels (16, 17, 19, 25, 26, 29, 31, 32).

To minimize variability and improve the accuracy of both clinical and research applications, we recommend that blood samples for AMH measurement be collected in the early follicular phase (typically cycle days 2–3), whenever feasible. At this time, estradiol levels are at their nadir, minimizing their suppressive effect on AMH secretion (33). Importantly, this is also the time window during which AMH measurements were obtained for the majority of participants in our model development and validation cohorts. For women with irregular menstrual cycles, identifying the early follicular phase may be more challenging. In such cases, clinicians should use careful cycle tracking and clinical judgment to approximate this window as closely as possible.

Repeat measurement of AMH may be considered in specific clinical scenarios, particularly when (i) the AMH value is unexpectedly low or high and inconsistent with other clinical findings (e.g., antral follicle count or reproductive history); (ii) the patient has recently undergone hormonal treatment (e.g., oral contraceptives, GnRH agonists); (iii) there is recent ovarian pathology (e.g., cysts, surgery, inflammation); or (iv) the measurement was taken during an ill-defined phase of the menstrual cycle or under unclear pre-analytical conditions. In such situations, a second AMH test—ideally performed in the early follicular phase and under stable physiological conditions—can improve result reliability and model prediction accuracy.

Longitudinal monitoring should be performed using the same validated assay platform. If switching platforms is unavoidable, calibration and conversion tools (such as our previously developed AMHConverter algorithm (34)) should be used to ensure comparability. Additionally, strict adherence to standardized protocols for sample collection, processing, transport, and storage is essential to minimize pre-analytical variability.

Finally, detailed clinical documentation—including the timing of the blood draw, menstrual cycle phase, concurrent medications, and any acute illness or environmental exposures—is crucial for interpreting AMH results accurately. By implementing these best practices, the robustness and clinical utility of AMH-based tools such as OvaRePred (HerTempo) can be further enhanced while maintaining consistency between model assumptions and real-world application.

### Challenges for high−reserve women

Our OvaRePred (HerTempo) tool assumes that women have already passed the plateau phase—typically reached during puberty—when ovarian reserve peaks, before predicting future reproductive milestones. Consequently, many younger women with a high ovarian reserve may exhibit similar ovarian reserve scores for many years, which poses challenges in accurately predicting the timing of future reproductive milestones. For example, in women whose ovarian reserve remains high and stable, the model’s predicted age of perimenopause onset may substantially precede the actual age at which it will occur.

### Platform flexibility and mitigation of batch variability

The updated version of the OvaRePred (HerTempo) tool now supports various AMH detection platforms, with conversion algorithms derived from our prior research (34). Moreover, we will continue to collaborate with reagent manufacturers to further mitigate the influence of batch variability on the results.

### Generalizability to the general population

The AMH–age model was developed from single-center ART data, yet our AMH distribution analysis shows that women under 40 in the community cohort have AMH values comparable to those in the ART cohort. This observation supports the model’s general applicability for women ≤40 years and provides reassurance that ART-derived AMH–age relationships mirror those in the general population. However, differences observed in women >40 years indicate that future studies should recruit more older general participants to improve accuracy across the entire age spectrum.

### Systemic effects of ovarian aging and holistic utility of OvaRePred

Ovarian aging is a pivotal component of the overall female aging process. Beyond its essential role in reproduction, the ovary functions as a crucial endocrine gland, regulating systemic homeostasis through the secretion of estrogen, progesterone, and androgens. As ovarian reserve diminishes and functional decline ensues, the consequent reduction in sex hormone production has been associated in prior literature with increased risk of cardiovascular diseases, osteoporosis, cognitive decline, metabolic disorders, immune dysregulation, and skin aging (35–43).

OvaRePred (HerTempo) was designed as an early−warning tool for ovarian aging. Specifically, for women with a low ovarian reserve score and an earlier predicted onset of perimenopause, OvaRePred may support earlier fertility planning or consideration of oocyte cryopreservation. Conversely, those with strong ovarian reserve and later predicted perimenopause may align reproductive timing with life goals. These fertility-focused applications remain the core validated purpose of the tool.

**Figure 5:**
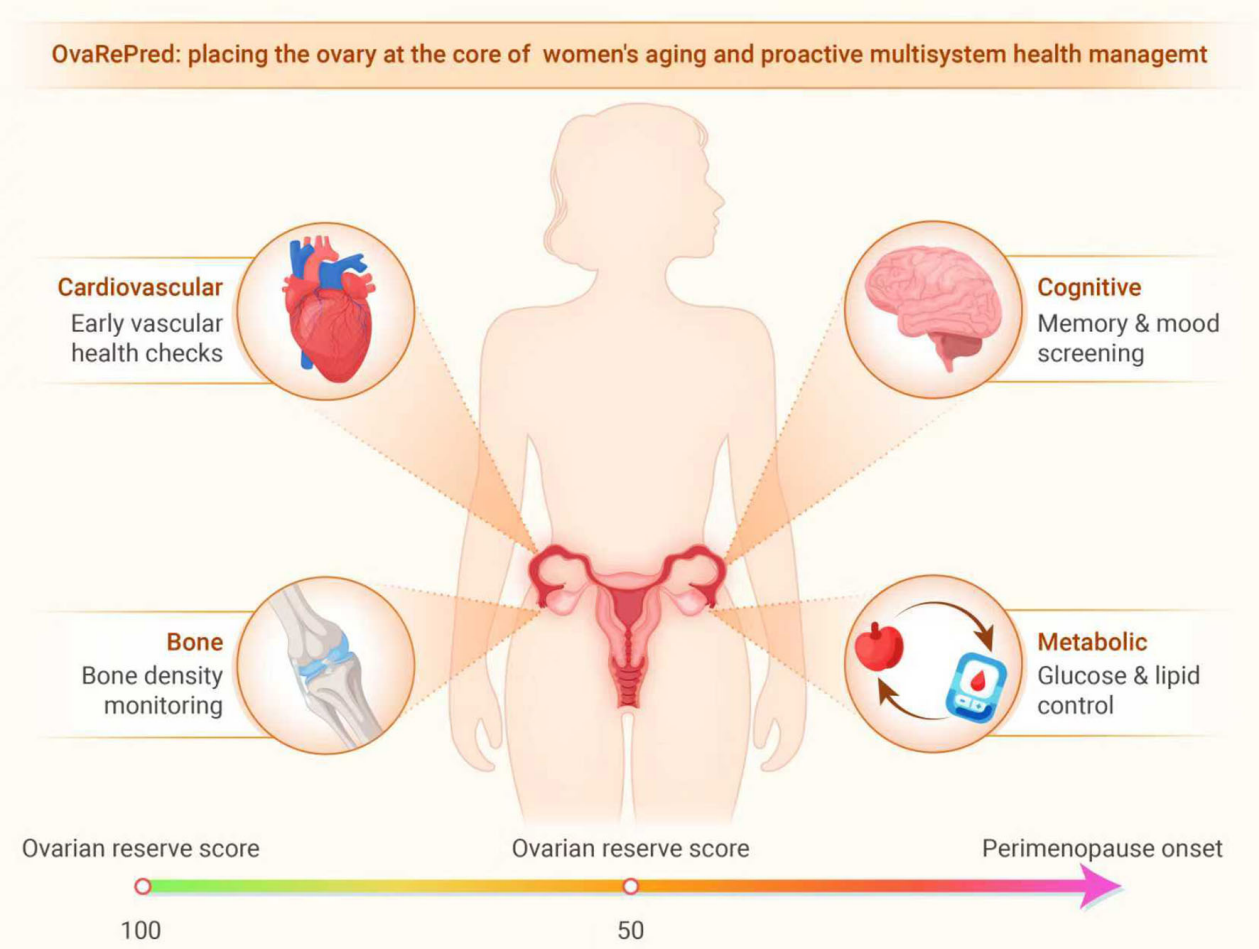
Infographic illustrating OvaRePred’s concept of ovarian reserve scoring. A female silhouette highlights the ovary as the “core of female aging,” linking endocrine age to cardiovascular, bone, cognitive and metabolic screening priorities. The gauge depicts reserve score (0‑100); a timeline aligns diminished reserve, perimenopause and menopause for anticipatory, proactive health management.

In addition to guiding fertility decisions, the OvaRePred tool can inform comprehensive health management, as illustrated in **Figure 5**. In this context, the significance of OvaRePred (HerTempo) extends far beyond predicting ovarian aging, offering a comprehensive tool for holistic women’s health management. However, the potential to expand OvaRePred’s utility for anticipatory health guidance remains an area for future research and should not be construed as a current clinical indication. While these associations suggest that ovarian health may play a broader role in women’s systemic well-being, we acknowledge that the current version of OvaRePred (HerTempo) has only been validated in the context of assisted reproduction. Therefore, these broader implications are only hypothetical future directions rather than validated applications.

### Population−level applications and public health integration

OvaRePred (HerTempo)’s validated, user-friendly algorithm enables scalable ovarian reserve assessment at the population level. Integration into national or regional women’s health programs could facilitate early detection of diminished ovarian reserve, guide fertility counseling, and inform public health resource planning. Key barriers—such as test cost, laboratory access, and low public awareness—can be mitigated through tiered screening approaches, point-of-care diagnostics, health education initiatives, and strategic policy support. Harnessing aggregated OvaRePred data may also enable population health surveillance and support data-driven policy-making to improve women’s health outcomes on a broader scale.

## Limitations

Despite significant progress achieved in this study, the OvaRePred (HerTempo) tool has several limitations that warrant further refinement. First, the current model is primarily developed based on single-center ART population data; thus, broader applicability requires additional samples and multi-center external validation. It is crucial to address variability across different ethnicities, regions, and cultural backgrounds to continuously optimize model parameters and improve both clinical utility and predictive accuracy. Second, the lack of documented blood collection timing (i.e., specific day of the menstrual cycle) for some reproductive-age women may affect result accuracy, underscoring the need for improved data collection protocols to ensure stability and reproducibility. Third, while temporal data splitting was used to simulate prospective validation, this approach may be subject to time-based confounding—such as changes in clinical protocols, shifts in patient population characteristics, or external factors like the COVID-19 pandemic—which could influence model performance. Future studies should consider combining temporal and random splitting strategies or incorporating external datasets to better mitigate such effects. Moreover, expanding the range of input parameters—such as ovarian aging variations under diverse disease conditions, and influences from medications, lifestyle factors, and environmental exposures—could enhance the tool’s adaptability and precision in complex clinical settings, supporting more personalized health management.

The fixed-interval hypothesis, which posits a consistent temporal relationship between reproductive aging events, underpins part of our predictive framework. Although it is supported by cross-sectional and historical population data, this assumption remains unvalidated at the individual level. Longitudinal confirmation would require tracking natural menstrual and fertility patterns over many years; however, in contemporary settings, such prospective designs are extremely difficult to implement. The widespread use of hormonal contraception, together with other medical or lifestyle interventions, obscures natural reproductive trajectories and greatly limits the feasibility of obtaining uninterrupted, long-term observations. Consequently, reliance on this unverified assumption constitutes a significant limitation, particularly for making individualized long-term predictions. Future research should prioritize the rare opportunity to conduct long-term, prospectively designed cohort studies in populations minimally influenced by hormonal contraception or other factors that alter natural reproductive aging.

Importantly, OvaRePred (HerTempo) focuses solely on quantifying ovarian reserve, reflected by follicle quantity, and does not directly assess follicle or oocyte quality, nor overall fertility potential. This distinction is critical in clinical scenarios such as PCOS, where patients may exhibit high follicle counts but impaired oocyte developmental competence. In such cases, a high reserve score does not necessarily equate to optimal reproductive capacity. Therefore, for a more comprehensive fertility evaluation, OvaRePred results should be interpreted alongside complementary diagnostic markers—such as oocyte maturity rates, detailed hormonal profiles, and, where available, indicators of oocyte or embryo competence. Integrating these multidimensional assessments enables a more nuanced understanding of reproductive potential and guides individualized clinical decision-making.

## Conclusion

In summary, OvaRePred (HerTempo), a comprehensive evaluation tool powered by big data and artificial intelligence, not only provides precise decision support for fertility planning but also paves a new path for holistic women’s health management. Through continual optimization and interdisciplinary integration, OvaRePred (HerTempo) is poised to become a vital bridge between reproductive medicine and overall health management, offering more precise and personalized health guidance for women.

## Data Availability

The original contributions presented in the study are included in the article/**Supplementary Material**. Further inquiries can be directed to the corresponding author.
