# Supplementary material for: OvaRePred (HerTempo): an enhanced ovarian aging clock for personalized reserve assessment, endocrine age modeling, and predicting reproductive milestones across the female lifecycle
Source: Front Endocrinol (Lausanne). 2025 Sep 12;16:1658068. doi: 10.3389/fendo.2025.1658068 (PMC12463964; doi:10.3389/fendo.2025.1658068)
Supplement: Supplementary file 1 [file DataSheet1.docx]

## AMH levels during ovarian stimulation

Although AMH levels remain relatively stable for most women over a single cycle, a subset exhibit significant intra‑cycle fluctuations. To assess how these variations impact predictive performance, we compared AMH measurements on cycle days 2 and 6 and on the hCG trigger day (approximately day 12) during ovarian stimulation in GnRH antagonist cycle, and built three different models using AMH and age, to identify the magnitude of AMH decline required to significantly compromise the model’s accuracy.

First, we applied a normality transformation to the non‑normally distributed AMH values (Figure 4A). Then, using a linear mixed‑effects model on the log‑transformed data, we found that, relative to AMH on cycle day 2 (AMH₂), AMH on day 6 (AMH₆) and day 12 (AMH₁₂) declined by approximately 17.4% and 49.7%, respectively (both p < 0.0001; Figure 4B), indicating that AMH levels peak in the early follicular phase and subsequently decrease markedly.

ROC curve analysis demonstrated that the model incorporating AMH on cycle day 2 plus age achieved the highest AUC (0.868; 95% CI, 0.814–0.908). Although its AUC did not differ significantly from that of the AMH on day 6 + age model (AUC = 0.860; 95% CI, 0.805–0.902), it was significantly superior to the AMH on day 12 + age model (AUC = 0.652; 95% CI, 0.584–0.715), as shown in Figure 4C. Moreover, confusion‑matrix metrics showed that the day 2 AMH + age model had the lowest misclassification rate (0.1099) and outperformed the others in sensitivity, F1 score, and Matthews correlation coefficient (MCC) (see Supplementary Material 1), indicating that this model is more accurate and reliable for predicting POR in women with large intra‑cycle AMH fluctuations.

In addition, during controlled ovarian stimulation, estradiol (E2) levels rise from the early follicular phase to a peak around day 12, and E2 is known to negatively regulate AMH^1^. Clinically, we have observed that some women—particularly those with endocrine disorders such as PCOS—exhibit substantial AMH variability within and between cycles, which can diminish the model’s predictive accuracy. Our findings suggest that using the highest measured AMH concentration for model evaluation is the most reasonable approach, as it corresponds to the time point at which predictive accuracy is maximized.

References:

1. Grynberg, M., Pierre, A., Rey, R., et al. (2012). Differential regulation of ovarian anti-mullerian hormone (amh) by estradiol through alpha- and beta-estrogen receptors. J Clin Endocr Metab **97**:E1649-E1657. DOI: 10.1210/jc.2011-3133.
